# Data on oxygen consumption rate, respiratory exchange ratio, and movement in C57BL/6J female mice on the third day of consuming a high-fat diet

**DOI:** 10.1016/j.dib.2016.02.066

**Published:** 2016-03-03

**Authors:** Phillip M. Marvyn, Ryan M. Bradley, Emily B. Mardian, Kristin A. Marks, Robin E. Duncan

**Affiliations:** University of Waterloo, Department of Kinesiology, Faculty of Applied Health Sciences, 200 University Avenue W., BMH1110 Waterloo, Ontario, Canada N2L 3G1

**Keywords:** Respiratory exchange ratio, VO2, Oxygen consumption, Physical, Activity, Mice, Fasting, High fat diet

## Abstract

Whole animal physiological measures were assessed following three days of either standard diet or high fat diet, in either the fasted or non-fasted states. Our data shows that acute 3-day high fat feeding increases whole body lipid oxidation. When this feeding protocol is followed by an overnight fast, oxygen consumption (VO_2_) in the light phase is reduced in both dietary groups, but oxygen consumption in the dark phase is only reduced in mice fed the high-fat diet. Furthermore, the fasting-induced rise in dark cycle activity level observed in mice maintained on a standard diet is abolished when mice are fed a high-fat diet.

**Specifications Table**

**Table:** 

Subject area	*Biology*
More specific subject area	*Animal Physiology*
Type of data	*Graph*
How data was acquired	*Comprehensive Laboratory Animal Monitoring System (CLAMS)*
Data format	*Means±S.E. from CLAMS data*
Experimental factors	*C57BL/6J female mice fed standard chow or high fat diet (45* *kcal% fat) for three days. Animals were assessed by CLAMS in either the fasted or non-fasted state.*
Experimental features	*Oxygen consumption (*VO_2_*), respiratory exchange ratio (RER), Physical Activity*
Data source location	*University of Waterloo, Waterloo, Ontario, Canada*
Data accessibility	*Data is provided within the article*

**Value of the data**

• These data are valuable to researchers interested in investigating the metabolic response to fasting
• These data are valuable to researchers interested in the physiology of acute high fat feeding
• These data are valuable to researchers studying female mice
• These data are valuable to researchers benchmarking physiological changes during different feeding conditions

## 1 Data

**Fig. 1:**
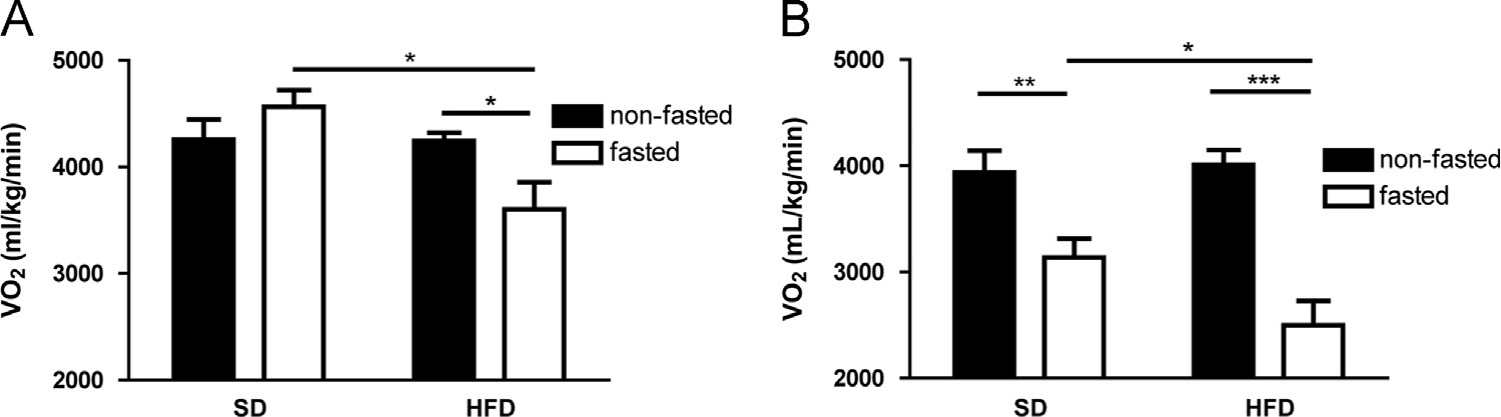
Data on oxygen consumption in fasted and non-fasted mice at day three of feeding a standard (SD) or high fat diet (HFD)**.** (A) VO_2_ during the dark phase. (B) VO_2_ during the light phase. Data are means±S.E.M. (*n*=7−9). (^*^P<0.05, **P<0.01, ***P<0.001).

**Fig. 2:**
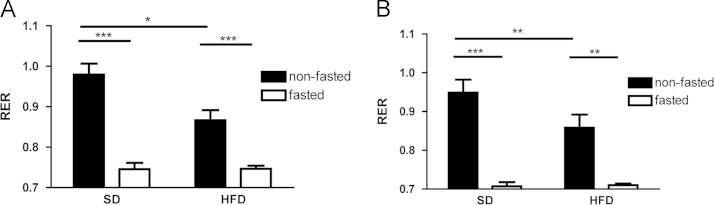
Data on the respiratory exchange ratio (RER) of fasted and non-fasted mice at day three of feeding a standard (SD) or high fat diet (HFD). (A) RER during the dark phase. (B) RER during the light phase. Data are means±S.E.M (*n*=7−9). (^*^P<0.05, **P<0.01, ***P<0.001, ****P<0.0001).

**Fig. 3:**
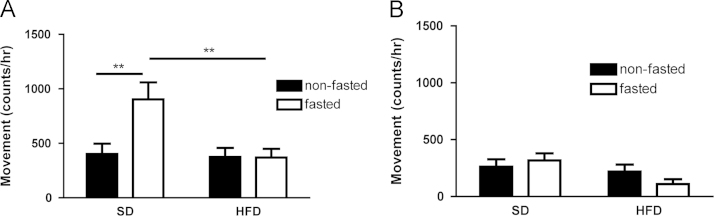
Data on activity of fasted and non-fasted mice at day three of feeding a standard (SD) or high fat diet (HFD). (A) Physical activity during the dark cycle. (B) Physical activity during the light cycle. Data are means±S.E.M (*n*=7–9). (^**^P<0.01).

Fasting during the light phase reduced oxygen consumption in female mice fed either a high-fat or standard diet (SD) for 3 days (Fig. 1B), but during the dark phase, fasting only reduced oxygen consumption when mice were fed a background high-fat diet (Fig. 1A). In both the dark and light phases, fasted mice fed a high-fat diet (HFD) had reduced oxygen consumption when compared to fasted mice fed SD (Fig. 1). Fasting significantly reduced the respiratory exchange ratio (RER) in mice fed either background diet, and consuming a HFD resulted in a lower non-fasted RER than consuming a SD, regardless of the phase of study (Fig. 2). Fasted mice fed a SD had increased activity during the dark cycle compared to non-fasted mice, although these data do not show a similar increase in activity during the dark cycle with fasting when mice are given a background HFD (Fig. 3A). During the light cycle, these data show no significant differences between dietary or fasting/non-fasting groups with regards to activity (Fig. 3B).

## 2 Experimental design, materials and methods

### 2.1 CLAMS

All animal procedures were approved by the University of Waterloo Animal Care Committee and were in accordance with the guidelines of the Canadian Council on Animal Care. Group-housed mice were maintained on a reverse light-dark cycle and fed a defined standard diet (SD) (Cat# D12450H (10 kcal% fat, 70 kcal% carbohydrate, 20 kcal% protein) from Research Diets, New Jersey, USA) for 1 week, prior to randomization to one of two groups. Half of the mice continued consuming D12450H, and these formed the SD group. Mice in the HFD group were instead provided with a diet containing 45 kcal% fat, 35 kcal% carbohydrate, and 20 kcal% protein (Cat#D12451 from Research Diets, New Jersey, USA). Soybean oil and lard provided the fat sources for the diets, while corn starch, maltodextrin and sucrose provided the carbohydrate source, and casein provided the protein source. Complete diet compositional data are available from the manufacturer. On the second day of feeding, mice were placed into individual cages in the Oxymax Comprehensive Laboratory Animal Monitoring System (CLAMS, Columbus Instruments) for an additional 24 h. Fasted mice had food withdrawn for ~12 h, from midway through the light cycle to midway through the dark cycle, and data shown are from these time periods for both fasted and non-fasted mice. The Oxymax system is an open-circuit indirect calorimeter for lab animal research allowing the measurement of oxygen consumption (VO_2_), respiratory exchange ratio (RER) and activity levels of mice. Oxygen consumption (VO_2_) is a measure of the volume of oxygen used to convert energy substrate into ATP. Respiratory exchange ratio (RER) is the ratio of carbon dioxide production (VCO_2_) divided by oxygen consumption, and can be used to estimate the fuel source for energy production based on the difference in the number of oxygen molecules required for the oxidation of glucose versus fatty acids [1]. An RER of 0.7 indicates that fatty acids are the primary substrate for oxidative metabolism, while an RER of 1.0 indicates that carbohydrate is the primary energy substrate [1]. Activity was calculated by summing the *X*-axis movement counts associated with horizontal movement. All protocols were approved by the University of Waterloo Animal Care and Use Committee.

### 2.2 Statistical analysis

The data are expressed as means±S.E.M. Statistically significant differences between two groups were assessed by one-way ANOVA. Significance is accepted at *P*<0.05.
